# Sugammadex in Emergency Situations

**DOI:** 10.3390/jpm13010159

**Published:** 2023-01-15

**Authors:** Cyrus Motamed

**Affiliations:** Departement of Anesthesia, Gustave Roussy Cancer Campus, 94080 Villejuif, France; cyrus.motamed@gustaveroussy.fr

**Keywords:** neuromuscular blockade, rocuronium, sugammadex, reversal, emergency situation

## Abstract

Sugammadex may be required or used in multiple emergency situations. Moderate and high doses of this compound can be used inside and outside the operating room setting. In this communication, recent developments in the use of sugammadex for the immediate reversal of rocuronium-induced neuromuscular blockade were assessed. In emergency surgery and other clinical situations necessitating rapid sequence intubation, the tendency to use rocuronium followed by sugammadex instead of succinylcholine has been increasing. In other emergency situations such as anaphylactic shock caused by rocuronium or if intubation or ventilation is not possible, priority should be given to resuming ventilation maintaining hemodynamic stability, in accordance with the traditional guidelines. If necessary for the purpose of resuming ventilation, reversal of neuromuscular blockade should be done in a timely fashion.

## 1. Introduction

**Figure 1:**
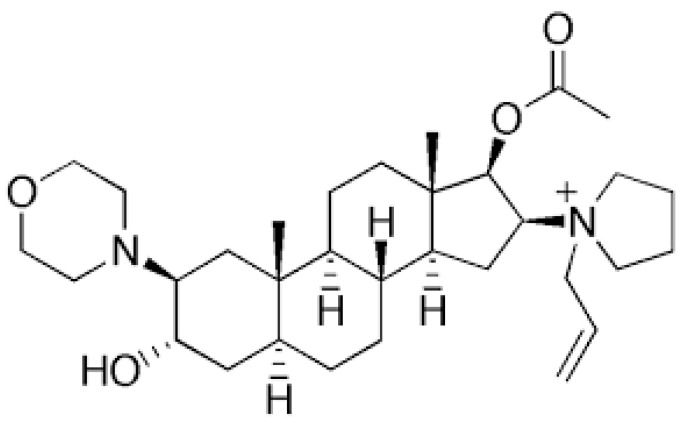
Chemical structure of rocuronium.

Muscle relaxation for rapid sequence intubation has traditionally been dominated by the use of succinylcholine, a depolarizing muscle relaxant with a rapid onset of action. However, it has a very short duration of action and causes several adverse events such as muscle pain, hyperkalemia, arrhythmia, and anaphylaxis, which have been known for several decades. In the last two decades, succinylcholine has been challenged by the non-depolarizing aminosteroid rocuronium (ORG 9426; Figure 1) and its specific neuromuscular blockade reversal agent, sugammadex (ORG 25969) [1].

Rocuronium bromide is an intermediate-acting non-depolarizing muscle-relaxant aminosteroid similar to vecuronium (ORG NC 45), which competitively binds to the nicotinic receptor in the neuromuscular junction and antagonizes the acetylcholine receptor.

**Figure 2:**
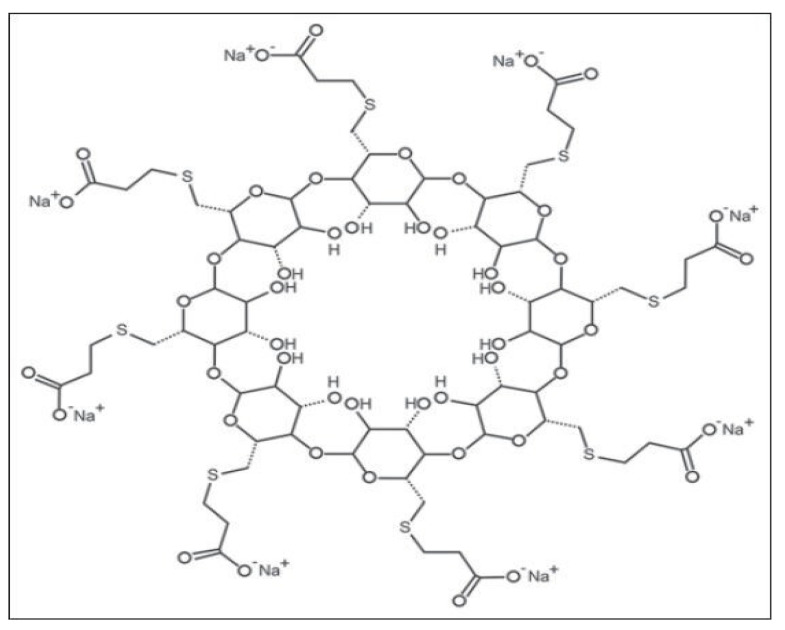
Chemical structure of sugammadex.

Sugammadex is a modified cyclodextrin designed to encapsulate non-depolarizing muscle-relaxant aminosteroid agents in their hydrophobic core, creating a 1:1 hydrophilic complex dependent on renal excretion (Figure 2). This complex inactivates the neuromuscular blocking agent and therefore effectively reverses profound neuromuscular blockade in humans. The elimination half-life in healthy adults is approximately 2 h, but this is significantly altered by renal impairment. Rocuronium and sugammadex are widely used in operating rooms and critical cares; however, this combination is now increasingly used in emergency situations in other settings such as emergency departments, out of hospital, and intensive care units. Therefore, all physicians in these specialties should be poised to acquire specific knowledge related to these molecules.

The chemical structure of sugammadex (S) is similar to a hollow cylinder, which can neutralize rocuronium (R) by encapsulation and creation of the RS complex.

Hydrophobic interactions encircle rocuronium within the cyclodextrin (sugammadex) cavity, resulting in the formation of a water-soluble complex.

This commentary aimed to update the current knowledge on this trend in the replacement of succinylcholine in potential emergency situations and other life threatening cases in which sugammadex could be used, such as in “cannot intubate, cannot ventilate (CICV)” situations, anaphylactic shock to rocuronium, and emergency neurological examination.

Situations were arbitrarily divided into operating room and non-operating room emergencies; however, most situations can take place in both settings. Specific comments on obese patients, pediatric patients, and the coronavirus disease (COVID-19) pandemic have been added. Studies included in this selective English literature review were published after 2012. The commentary included meta-analyses, randomized trials, and observational and expert opinions registered in the electronic databases Medline and Google Scholar. When recent information about a specific subject was not available, older references were used.

## 2. Operating Room Emergencies

### 2.1. Rapid Sequence Induction

The aim of rapid sequence induction (RSI) in general anesthesia is to protect the airway with rapid endotracheal intubation from the aspiration of gastric content to permit surgery. RSI is defined as preoxygenation followed by rapid induction of general anesthesia and muscle relaxation and immediate attempted endotracheal intubation. In modern anesthetic practice, propofol and rocuronium are increasingly used for RSI. If rocuronium is used in the situation of “cannot intubate cannot ventilate”, a higher dose of sugammadex can quickly reverse neuromuscular blockade. Although RSI was originally described in the operating theater, it is no longer the only place where RSI is performed. Additional places are emergency departments, intensive care units, and prehospital settings. Propofol has mild muscle relaxant properties [2]; however, in other settings, other agents such as ketamine, thiopentone, and etomidate are used. Adequate muscle paralysis obtained with a neuromuscular blocking agent is a major factor to obtain better laryngoscopic conditions in this context to avoid failed intubation. By definition, RSI per se is mostly an emergency situation in which airway control must succeed, as alternative methods such as awakening are not an options. 

A Cochrane-based systematic review conducted in 2017 compared rocuronium and succinylcholine and described that succinylcholine-induced blockade obtained more excellent intubation conditions overall than did rocuronium, while thiopental was used as an induction agent [3]. However, anesthesiologists extensively use propofol as an induction agent. Etomidate is also heavily used in emergency departments, but nowadays, intubation conditions tend to be similar with lesser or even no more use of thiopental and/or etomidate.

The use of rocuronium followed rapidly by sugammadex in adult patients for elective surgery requiring RSI, which in fact can be partially assimilated to emergency situations, has been reported to be safe and resulted in earlier return to spontaneous ventilation compared with succinylcholine alone [4]. In a strong randomized controlled study conducted by Sorensen et al., one of sixty-one patients had a difficult intubation, which was subsequently withdrawn. A similar combination was also reported to be effective in obstetric patients [5]. However, the safety of sugammadex in non-cesarean surgery for obstetric patients has not been fully elucidated [6], although the results of recent studies have been encouraging [7,8]. In addition, for failed intubation, the persistence of paralysis with rocuronium might help promote a supplemental new technique such as video-laryngoscopy, which is easier to perform while the patient is still paralyzed. Nevertheless, RSI protocols significantly vary both within and between countries [9], but profound muscle relaxation is generally admitted with both succinylcholine and rocuronium at an adequate dosage of 1–1.2 mg/kg, which provides good intubation conditions. Succinylcholine-induced blockade can recover generally after 6–12 min, but anticholinesterase deficit always confers a minimum risk of prolonged recovery. On the other hand, if necessary, rocuronium blockade may be reversed with sugammadex 16 mg/kg within 3 min after injection, which should be available and prepared rapidly if needed in some circumstances. Currently, the trend continues to favor rocuronium followed by sugammadex [10], as this combination has been reported to have less adverse effects, with a unique possibility of the rapid reversal (3 to 4 min) of profound neuromuscular paralysis with 16-mg/kg administration.

While the onset time of action of both drugs (rocuronium 1.2–1.5 mg/kg, succinylcholine 1 mg/kg) are close to 60 s, spontaneous recovery favors mostly succinylcholine (45 min vs 11 min, depending on several factors). In addition, rocuronium 0.6 mg/kg can be significantly potentiated by molecules such as magnesium. For example, using a standard dose of rocuronium can significantly shorten the onset (72 sec) and prolong the duration (+35%) [11]; however, this implies a possible quicker recovery in case of sugammadex reversal since less rocuronium might be administered in case of RSI.

The major adverse events are anaphylaxis mostly related to succinylcholine administration. Hyperkalemia, prolonged paralysis, arrhythmia, myalgias, fasciculations, and triggering malignant hyperthermia are mostly due to succinylcholine. In addition to rocuronium anaphylaxis, allergic reactions were also reported for sugammadex [12], while hemodynamic instability was reported for rocuronium and sugammadex [13].

### 2.2. CICV Situations

The introduction of sugammadex, with its rapid reversal of even profound neuromuscular blockade, has led to the suggestion that it is a potential rescue strategy in CICV situations. However, because of the nature of this emergency, a real comparative randomized study protocol is impossible. Several cases have been described [14] in addition to studies simulating CICV cases. The latter can only simulate facts or predict results because they are not real-life situations [15]. Some of these simulations predicted that obese and morbidly obese patients may become hypoxic before intubation. For example, after 3 min of preoxygenation, only morbidly obese patients might become hypoxic [16]. In addition, the time to achieve a respiratory rate of less than four per minute after reversal with sugammadex may be as long as 12 min in 5% of all types of patients. Nevertheless, case reports of effective rescue in neonates who successfully recovered from a CICV situation without tracheostomy have been published [17]. Additional consideration should include the preparation time and sufficient dosage of sugammadex in the operating room [15,18]. Cost issues rule out pre-drawn syringes, while manikin simulations predicted that the time to prepare a high dose (16 mg/kg) of sugammadex is approximately 6–7 min; however, a high dosage may potentially add other adverse events such as bradycardia and asystolia [16]. Nevertheless, adequate contemporary attitudes are still in favor of prioritizing airway management, with a focus on oxygenation and ventilation rather than pharmacological initiatives [14,19,20]. Muscle relaxants are considered to facilitate ventilation, but after prompt reversal, spontaneous ventilation is not guaranteed [18]. Moreover, the restoration of full muscle tone could endanger the situation and lead to opposite efforts to ventilate the patient [21].

### 2.3. Anaphylaxis

Rocuronium-induced anaphylaxis is the second most important cause of anaphylaxis among muscle relaxants, after succinylcholine [15]. After several case reports of improved clinical status after the administration of sugammadex, it was initially hoped that rocuronium-induced anaphylaxis could be reversed at least partially by sugammadex [22,23,24]. Most contemporary guidelines on the management of anaphylaxis focus mainly on drugs such as epinephrine and other fluid resuscitation approaches to restore appropriate cardiac output [19,20]. In addition, some case reports addressing the failure of sugammadex to restore adequate hemodynamics have been reported [25].

Regarding anaphylaxis induced by sugammadex, it has now been clearly confirmed [12,26] that this drug alone can cause anaphylactic reactions, with an estimated incidence of approximately 1 per 300 persons in a 20,000 cohort population [27]. This reaction is not dose dependent, and the efficiency and reliability of the reversal should not be overridden by the risk of anaphylaxis [28], which is probably higher than that with neostigmine [29].

In addition, the sugammadex-rocuronium complex CRS has been reported to promote anaphylactic reactions [30,31,32], but this needs further investigation. Anaphylaxis has been suggested to result from the use of sugammadex complexes with rocuronium only and not from the use of sugammadex or rocuronium alone, which indicates that drug antigenicity may be modified during CRS formation [33].

### 2.4. Emergency Situations in Obese Patients 

Obese patients are at increased risks of aspiration with pathology such as hiatal hernia or delayed gastric emptying due to autonomic neuropathy (diabetes mellitus); however, for those patients with no such conventional risk factors RSI should not be a routine practice. Body mass index of greater than 50 kg·m^−2^ is an independent predictor of both difficult intubation and face mask ventilation [34]. In case of RSI for an inadequate period of fasting or abdominal pathology (rocuronium 1.2 mg/kg), ideal body weight IBW for muscle relaxation followed by sugammadex 8–16 mg/kg actual body weight permit a better control of neuromuscular block in case of failed intubation [35]. This scenario requires enough sugammadex available on site before induction. Indeed, using sugammadex with IBW delays moderate and deep neuromuscular blockade recovery [36]; unfortunately, data are not available for immediate reversal in obese patients. Here again, if ventilation is impossible, other means of adequate oxygenation must be considered and phamacological rocuonium reversal is not a priority. 

## 3. Non-Operating Room Emergencies

The use of rocuronium at 1 or 1.2 mg/kg (RSI) has been increasing in non-operating room emergency contexts; however, the timing and degree of reversal are still controversial [37].

The use of rocuronium and sugammadex when needed in a prehospital setting was suggested in 2010 [38]. The use of the rocuronium followed rapidly with sugammadex has been increasing [37] mostly among those with experience with this drug. As non-anesthesiologists are increasingly involved in RSI in the non-operating room environment, this reversal approach is expected to increase significantly.

In the emergency department setting, rocuronium 1 mg/kg was as successful as succinylcholine for first-attempt emergency intubation, but succinylcholine had more contra indications such as hyperkaliemia, known cholinesterase deficiency, recent burns, or muscle myopathies [39]. These results have also been confirmed in a French multicentric randomized trial for out-of-hospital emergency intubations [40]. In this study, as part of the protocol, rapid reversal with sugammadex for rocuronium muscle relaxation was permitted. In one case, sugammadex was used almost immediately after a failed intubation, but the patient did not wake up earlier than 45 min because of status epilepticus, and the physician did not follow the recommended algorithm for difficult intubation.

### Rapid Arousal for Neurological Assessment

In general, patients requiring neurological assessment should not be paralyzed except for emergency intubation situations such as status epilepticus, in which rapid reversal by rocuronium and sugammadex would be useful.

Several case reports on this particular indication have been published. Although these have been classified as non-operating room emergencies, neurological assessments can also be performed in operating room contexts, such as after traumatic or non-tra-umatic brain [38] and spine injury [41]. High doses of sugammadex can assist in facilitating a timely neurological examination because muscle paralysis recovery can be obtained within minutes [42].

In a retrospective study conducted in an emergency department, no difference in hemodynamic instability was observed between the patients who received sugammadex and those who received neostigmine for reversal [43]. In a case report, it has been hypothesized that the direct effect of high-dose sugammadex on the central nervous system is arousal from propofol anesthesia, but no further concrete investigations were made [38].

## 4. Pediatric Emergencies

Pediatric airway emergency complications (1970 through 1990) accounted for up to 36% of reported American Society of Anesthesiologists closed claims, although with a significant decreasing trend [44], it still implicates extreme vigilance in the dosing and manipulation of muscle relaxants and reversals.

The pharmacodynamics/pharmacokinetics and safety profiles of rocuronium and sugammadex are almost similar between children and adults, and an increasing number of studies have described their efficacies in pediatric patients of all ages. Decreased heart rate has been reported in children after sugammadex administration. Nevertheless, indications remain similar to those in adults, such as rapid neurological assessment. Once again, extreme caution is warranted in CICV situations [42]. A case report of the use of sugammadex at a dose of 16 mg/kg in an 850-g neonate in a CICV situation was reported to be effective [16]. This report, together with other sparse data of successful treatment with sugammadex in CICV situations in children, still does not support the use of sugammadex in that particular emergency situation, as the duration of apnea can last for approximately 15 min in 5% of patients [45]. In the United States, the use of sugammadex in children was only introduced in 2016 [46].

## 5. Emergency Intubation after Sugammadex Administration

Emergency intubation and neuromuscular paralysis after sugammadex administration are better performed with benzylquinolones. Rocuronium can be used, but its pharmacological effect, either its onset or offset, could be compromised because of the presence of sugammadex molecules in the bloodstream, as sugammadex is only excreted in urine, with a rate of clearance equivalent to the glomerular filtration rate.

In a study with healthy volunteers, Cammu et al. [47] suggested an inverse relationship between the onset and the time interval between sugammadex administration and additional rocuronium injection. A 30 min waiting time after sugammadex reversal appeared to be the cutoff to decrease the onset time to less than 2 min if an RSI dose of 1.2 mg/kg for rocuronium is used. Finally, succinylcholine can also be indicated in this context.

## 6. COVID-19 Pandemic

To intubate COVID-19 patients in an acute respiratory distress phase or in a scheduled surgical procedure, most societies have suggested a modified RSI technique to minimize the likelihood of aerosol generation. The suggested pharmacological plan in this context is rocuronium administration (1.2–1.5 mg/kg) followed 3 min later by the administration of sugammadex 16 mg/kg to reverse muscle relaxation quickly if necessary [48]. However, muscle relaxant administration might also be necessary for positioning patients with refractory hypoxia in prone position. Whether in critical care, operating room, or in or out of hospital, intubated COVID-19 patients might be considered as emergency situations because of their critical respiratory prognosis [49]; therefore, an adapted dosage of sugammadex in case of emergency reversal should be available. 

## 7. Monitoring Neuromuscular Blockade

The clinical assessment of paralysis induced by a neuromuscular blocking agent is not reliable enough; therefore, for the sake of accuracy, instrumental quantitative monitoring of muscle paralysis is widely recommended or even mandatory in some countries. This is mostly performed by measuring the evoked response of the adductor pollicis muscle of the thumb after four consecutive supraphysiological stimulations (30–40 mA) via the corresponding ulnar nerve. Four twitch responses are labeled “train of four,” and the amplitude of the fourth response compared with the first is the train of four (TOF) ratio.

Devices with a quick setup without calibration may be more suitable for rapid sequence or other emergency situations [50]. During full recovery, for a safe extubation, a ratio higher than 90% is necessary to guarantee that no residual paralysis complications would occur. The “train of four” stimulation with quantitative assessment is now somehow mandatory according to several international guidelines [51]. Different methods of stimulations are now available, including accelerography, kinemyography, and electromyography. If quantitative monitoring is not available (e.g., in emergency departments), it is still better to use a simple nerve stimulator or clinical assessment alone.

Post-tetanic count stimulation is another mode of stimulation for assessing deep neuromuscular blockade. In this configuration, a tetanic stimulation (50 Hz) is generated for 5 sec, and the evoked responses to a single twitch stimulation are counted. The higher the number of detected responses during the post-tetanic count is, the sooner normal TOF responses return.

It should be emphasized that the objective of neuromuscular monitoring is, first, to prevent postoperative paralysis using the most effective dose and, second, to prevent future interference by injecting the lowest effective dose in case muscle paralysis is needed urgently.

The recommended doses of sugammadex with regard to the results of neuromuscular monitoring are as follows: four weak twitches on TOF, 1 mg/kg; reappearance of the second of four twitches, 2 mg/kg; no twitch on TOF but with one–two post-tetanic count stimulations, 4 mg/kg; immediately after injection (rocuronium 0.6 or 1.2 mg/kg), 16 mg/kg; patients aged 2 through 17 years (shallow block), 2 mg/kg.

With regard to sugammadex administration, adequate monitoring may avoid recurarization. While monitoring may not be readily available in an emergency setting, clinicians may use clinical assessment and expected pharmacokinetics/phamarcodynamics properties of rocuronium or other relaxants until the clinical situation stabilizes; however, objective monitoring remains the only method to confirm adequate recovery.

## 8. Conclusions

Sugammadex remains an agent for reliable pharmacological reversal in routine clinical practice; however, in emergency situations such as CICV or anaphylaxis, the primary focus should be the restoration of the airway and/or hemodynamics because alternative approaches could significantly endanger the patient’s life. In emergency situations necessitating RSI more and more practitioners are using rocuronium followed by sugammadex when rapid reversal is indicated.

## Data Availability

Not applicable.
